# Supplementary material for: Perceptions and Expectations of Academic Staff in Bucharest towards the COVID-19 Pandemic Impact on Dental Education
Source: Int J Environ Res Public Health. 2023 Jan 18;20(3):1782. doi: 10.3390/ijerph20031782 (PMC9914722; doi:10.3390/ijerph20031782)
Supplement: Supplementary file 1 [file ijerph-20-01782-s001.zip › ijerph-2143096-supplementary.pdf]

Table S1. Questionnaire on the perception level of university teachers regarding the impact of the COVID-19 pandemic on dental education.

Question	Abbreviation	Questions and Romanian translations (italic)
**Q1**	Age	Age<br>*Vârsta*
**Q2**	Gender	Sex<br>*Sexul*
**Q3**	Academic career duration	Career duration in higher medical education<br>*Vechimea în învăţământul superior medical*
**Q4**	The academic year of teaching	What year/years of study do you teach?<br>*La ce an/ani de studiu predați?*
**Q5**	Academic degree	Academic degree in higher medical education<br>*Gradul didactic în învăţământul superior medical*
**Q6**	Infection with SARS-CoV-2	Have you been infected with SARS-CoV-2?<br>*Aţi trecut prin infectarea cu SARS-CoV-2?*
**Q7**	Emotionally affected	Has the COVID-19 pandemic affected you hard emotionally?<br>*Pandemia de COVID-19 v-a afectat puternic din punct de vedere emoțional?*
**Q8**	Stress impact	Did the stress you felt triggered by the COVID-19 pandemic have impact on your teaching performance?<br>*Stresul declanșat de pandemia de COVID-19 resimțit de dumneavoastră a avut impact asupra propriei performanțe de predare?*
**Q9**	Sleep quality	Has your sleep quality decreased with the outbreak of the COVID-19 pandemic?<br>*Calitatea somnului dumneavoastră a scăzut odată cu declanșarea pandemiei de COVID-19?*
**Q10**	Anxiety	After returning to on-site education with the students by adopting the hybrid system, has the feeling of anxiety about the possibility of your SARS-CoV-2 infection increased?<br>*După revenirea la învaţământul faţă în faţă cu studenţii prin adoptarea sistemului hibrid, a crescut sentimentul de anxietate cu privire la posibilitatea infectării cu SARS-CoV-2?*
**Q11**	Technodifficulties	Has the transition to online education through digital platforms imposed by the lockdown measures in the COVID-19 pandemic occurred for you without technological difficulties?<br>*Tranziţia la învăţământul online prin intermediul platformelor digitale, impusă de măsurile de lockdown în cadrul pandemiei de COVID-19 s-a produs în ceea ce vă priveşte fără dificultăţi tehnologice?*
**Q12**	Difficulties in transmitting academic information	Did you encounter major difficulties in learning to transmit academic information through digital platforms during the COVID-19 pandemic?<br>*Deprinderea transmiterii informaţiei academice prin intermediul platformelor digitale în timpul pandemiei de COVID-19 de către dumneavoastră a întâmpinat dificultăţi majore?*

**Q13**	Quality of the academic information	Did the quality of the academic information to students in a fully online system suffer during the restrictions imposed by the COVID-19 pandemic?<br>*Calitatea informaţiei academice studenţilor în sistem integral online în timpul restricţiilor impuse de pandemia de COVID-19 a avut de suferit?*
**Q14**	Difficulty of objective assessment of students	Did the evaluation of students in the online format imposed by the COVID-19 pandemic encounter significant problems, such as the difficulty of objective assessment of them, respectively the impossibility of preventing fraud attempts and frauds?<br>*Evaluarea studentilor in formatul de tip online impus de pandemia de COVID-19 a întâmpinat probleme semnificative, precum dificultatea aprecierii obiective a acestora, respectiv imposibilitatea prevenirii tentativelor de fraudă şi a fraudelor?*
**Q15**	Acquiring practical manual skills by using traditional simulators	Considering the COVID-19 pandemic, can the acquisition of practical manual skills by dental students be successfully supported by the use of traditional simulators, working models, etc., depending on the specifics of each clinical specialty?<br>*Având în vedere pandemia de COVID-19, deprinderea abilităţilor manuale practice de catre studenţii de medicină dentară poate fi susţinută cu succes prin utilizarea simulatoarelor tradiţionale, a modelelor de lucru, etc, în funcţie de specificul fiecărei specialităţi clinice?*
**Q16**	Acquiring practical manual skills by using haptic devices or virtual reality	Are simulation systems, such as haptic devices or virtual reality supported by e-learning platforms, the optimal solution for the acquisition of practical manual skills by dental students, during and after the COVID-19 pandemic?<br>*Sistemele de simulare, precum dispozitivele haptice sau realitatea virtuală sustinute de platformele de e-learning sunt soluţia optimă pentru dobândirea abilităţilor manuale practice de catre studenţii la medicină dentară, în timpul, cât şi ulterior pandemiei de COVID-19?*
**Q17**	Effects of digital and/or virtual dental education	Did the replacement of traditional dental medical education with digital and/or virtual ones during the lockdown period as a result of the COVID-19 pandemic bring more favorable effects than disadvantages to the educational system?<br>*Inlocuirea învăţământului medical stomatologic tradiţional cu cel digital şi/sau virtual în timpul perioadei de lockdown ca urmare a pandemiei de COVID-19, a adus mai multe efecte favorabile decât dezavantaje sistemului educaţional?*
